# Experimental demonstration of a new near-infrared spectroscopy technique based on optical dual-comb: DC-NIRS

**DOI:** 10.1038/s41598-023-37940-1

**Published:** 2023-07-05

**Authors:** Roberto Barreiro, Frank Sanabria-Macías, Julio Posada, Pedro Martín-Mateos, Cristina de Dios

**Affiliations:** 1 Arquimea Research Center, Quantum Technologies, 38320 San Cristobal De La Laguna, Tenerife Spain; 2 University Carlos III of Madrid, SITe Group, 28911 Madrid, Spain

**Keywords:** Near-infrared spectroscopy, Frequency combs, Near-infrared spectroscopy, Optical imaging

## Abstract

We present a novel near-infrared spectroscopy technique based on Dual-Comb optical interrogation (DC-NIRS) applied to dispersive media. The technique recovers the frequency response of the medium under investigation by sampling its spectral response in amplitude and phase. The DC-NIRS reference and sample signals are generated using electro-optic modulation which offers a cost-effective, integrable solution while providing high adaptability to the interrogated medium. A careful choice of both line spacing and optical span of the frequency comb ensures that the retrieved information enables the reconstruction of the temporal impulse response of the medium, known as the diffuse-time-of-flight (DTOF), to obtain its optical properties with a 70 µs temporal resolution and 32 ps photon propagation delay resolution. Furthermore, the DC-NIRS technique also offers enhanced penetration due to noiseless optical amplification (interferometric detection). The presented technique was demonstrated on a static bio-mimetic phantom of known optical properties reproducing a typical brain’s optical response. The DTOF and optical properties of the phantom were measured, showing the capabilities of this new technique on the estimation of absolute optical properties with a deviation under 3%. Compared to current technologies, our DC-NIRS technique provides enhanced temporal resolution, spatial location capabilities, and penetration depth, with an integrable and configurable cost-effective architecture, paving the way to next-generation, non-invasive and portable systems for functional brain imaging, and brain-computer interfaces, among other. The system is patent pending PCT/ES2022/070176.

## Introduction

The steady progression and refinement of Near-Infrared (NIR) spectroscopy over the past decades have been a cornerstone in fields such as chemistry^1^, biology, and medicine^2^. This well-established technique has seen a significant evolution, from vibrational spectroscopy to the study of biological tissues. The advancements in new optical technologies in the NIR spectrum have further bolstered this development, enabling more precise and diverse applications. This progression has led to the emergence of NIRS, an in-vivo spectroscopic technique that allows for the non-invasive study of living tissue activity, specifically brain hemodynamics^3^.

The early study published by Jörbis^4^ in 1977 demonstrated that near-infrared radiation allows for sufficient photon transmission through organs for the monitoring of cellular and vascular events, by the measurement of hemoglobin concentrations. These findings inaugurated NIRS as a technique to exploit the infrared window to study brain metabolism, physiology, blood flow, or localized neural activity^3,5^ thanks to its capacity to measure brain hemodynamics. When neurons respond to stimuli, they increase their metabolic activity, which leads to higher oxygen consumption. This process transforms oxyhemoglobin (HbO) into deoxyhemoglobin (HbR), thereby altering the optical properties of the tissue within a spatial range of a few millimeters to centimeters. These changes can be measured by illuminating the area with NIR radiation and analyzing the transmitted beam, thus obtaining information on localized neural activation in the brain^6–8^.

However, assessing the changes in the optical properties of biological tissues induced by hemodynamics (primarily absorption, $\mu _a$, and scattering, $\mu _s$, at a fixed wavelength) involves measuring the absorption and propagation delay of the transmitted light, which is a challenging task due to the strong attenuation and dispersion they exhibit. This scenario motivated theoretical advances to understand the propagation of NIR radiation in living tissues^9^ and also gave birth to a variety of experimental techniques capable of overcoming these initial drawbacks and turning them into strengths^2,3^.

There exist different classes of NIRS techniques, which differ in the optical interrogation, and the type and quality of the information they retrieve^10,11^. Nowadays, the main three techniques that allows the retrieval of optical properties in turbid media are known as continuous wave (CW), frequency-domain (FD), and time-domain (TD). CW-NIRS extracts only relative changes in the optical properties of the biological tissue while being illuminated by a constant power of infrared light. It is a cost-effective portable technique, but the spatial information that it features is mainly topological unless a high density of optodes (HD-DOT)^12^ and heavy processing is employed. FD-NIRS, on the other hand, retrieves the amplitude and phase of an intensity-modulated optical source^13^. This extra information allows for the estimation of the absolute value of the optical properties and blood oxygen concentrations^13^ in the tissue. The technique is not as straightforward and cost-effective as CW and its depth discrimination capabilities are limited in comparison with the third mainstream approach, time-domain (TD). TD-NIRS studies the shape of a transmitted short pulse (ps to fs duration) in order to reconstruct the DTOF or impulse response^14^ of the medium. The DTOF gives information about the optical paths inside the medium, which is crucial to localize perturbations and inferring the inherent optical properties within the tissue. TD can recover such information at the cost of a complex implementation that includes ultrashort pulsed-optical sources and photon counting detectors^15^, which limits scalability. The spatial resolution of these techniques is dependent upon the specific method implemented, the placement of the probe, and the distance between the light source and detector. Generally, it spans from 1 to 4 cm, with advanced methods like TD-NIRS and HD-DOT capable of achieving a higher resolution. While these techniques are well established and commercial solutions based on them are already in the market, a new technique relying on a tunable interferometric scheme has been recently proposed. Interferometric NIRS (iNIRS)^16^ sweeps the wavelength of the interrogating light and mixes it with a reference arm to recover DTOF-related information. An early demonstration of the technique retrieves the optical properties of a medium and the dynamics of the scatterers present in it^17^.

NIRS is an evolving field open to new techniques that can fill the gaps left by current approaches. The present work shows how Dual-Comb spectroscopy^18^ (DCS) can contribute to upgrading the potential of NIRS. DCS is based on the multi-heterodyne detection of two optical frequency combs (OFC) with slightly different frequency spacings^19^. An OFC consists of a series of discrete, equally spaced frequency lines, serving as a precise spectral ruler for high-resolution, ultra-stable measurements.^20^ Dual-Comb (DC) spectroscopy offers a fast, precise, parallel optical interrogation of the frequency response of a sample without the need for moving parts^18^. Moreover, DCS technique downconverts optical frequencies into the radio-frequency (RF) domain, making it a straightforward task to acquire and process the spectral information it recovers. In many implementations, DCS employs OFC generated using mode-locked lasers^21^, but electro-optical OFC techniques offer a modular solution with configurable resolution and spectral range^22,23^. Electro-optic DCS uses a single optical seed to generate both OFCs, which translates into an intrinsic synchronization between the pair of OFCs, hence providing enhanced phase and amplitude stability^24^. Although DCS systems have demonstrated their remarkable capabilities from the visible to the MIR and THz windows in several fields such as metrology, vibrational spectroscopy, or ranging^21^, there is no current demonstration of their usefulness when it comes to dispersive or turbid media, which is the area in which NIRS excels, since scattering seriously damages the coherence needed between the OFC arms of DCS systems. Only single OFC combined with CCD cameras and 2D spatial imaging^25,26^ have been reported, but there is a clear niche in the use of this technique for NIRS.

In this work, we present the first experimental results demonstrating a new NIRS interferometric technique based on dual-comb (DC-NIRS) interrogation of highly dispersive media^27^ that is capable of retrieving absolute optical properties and DTOF information from the sample under study. The use of DCS allows for a parallel sampling of the amplitude and phase of the frequency response of highly dispersive media. Despite the loss of coherence between the OFC arms of the DC-NIRS system in dispersive samples, we demonstrate that it is possible to reconstruct the temporal impulse response of the medium or DTOF. The temporal resolution and range of the measurements of the DTOF can also be configured in an agile way by adjusting the number and the frequency spacing of tones in the optical combs. Our results show how the new DC-NIRS technique interrogates a calibrated phantom whose optical properties reproduce those of the human brain. DC-NIRS is comparable to TD and FD systems and it allows for absolute optical characteristics and should be a viable solution to perform hemoglobin concentrations evaluation while recovering DTOF information for a depth-resolved functional analysis. The parallel nature of the interrogation allows for a DTOF evaluation with a sub-ms resolution and an increase of the overall penetration due to the interferometric noiseless optical amplification.

## Methods and Materials

### Recovery of optical properties and depth information of dispersive media

The DC-NIRS technique we present can overcome the main challenge faced by interferometric techniques when interrogating dispersive media, this is, how to recover spectral information with sufficient quality when the high scattering sample limits the mutual coherence between the sample and reference optical signals^27^. We demonstrate that partial coherence between these signals is sufficient for the Dual-Comb technique to sample the frequency response of the media in phase and amplitude.

**Figure 1:**
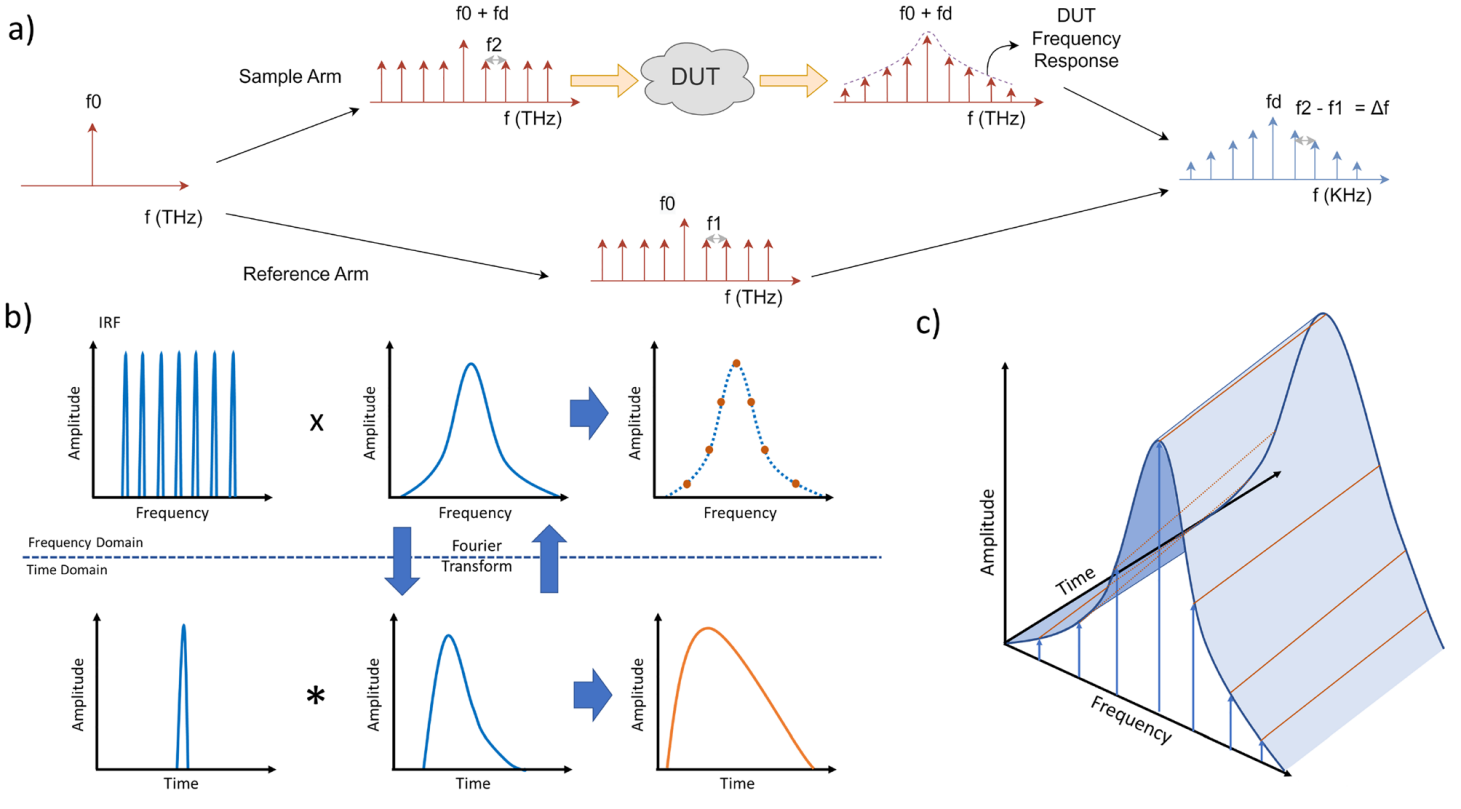
(**a**) The measurement principle of the system is based on sampling the impulse response of the medium through a set of evenly-spaced optical frequencies using as a reference another coherent optical comb with slightly different frequency spacing. The use of a carefully adjusted reference allows for a down-conversion of optical frequencies to RF domain, hence, simplifying the acquisition of the information in the latter domain due to the broad catalog of detection and acquisition techniques. (**b**) DC-NIRS works in the frequency domain. Its operation can be described as a multiplication of the medium’s frequency response by a set of evenly-spaced frequencies. In the time domain, measurements of the diffuse time of flight (DTOF) are based on a convolution of a short pulse or Instrument Response Function (IRF) with the impulse response of the medium. (**c**) Since the frequency response is sampled at the frequencies of the sample OFC for short time intervals, DC-NIRS allows the evaluation of the temporal evolution of the frequency response.

DC-NIRS is based on the interferometric mixing of two OFC signals (Fig. 1a) generated from a single continuous wave laser source via electro-optic (EO) modulation^28^. The sample OFC, which is composed of equally-spaced lines separated $f_2$ and shifted a small frequency $f_{d}$ in the kHz or MHz range, enters the tissue and is interferometrically mixed with the reference comb with a frequency spacing of $f_1$. Both repetition rates are related as $f_2 = f_1 + \Delta f$. Thus, the reference and sample OFCs of the DC-NIRS can be expressed as:

1 $$\begin{aligned} E_{ref}(t) \propto \sum _{m_1=-M_1}^{M_1} A^{(1)}_{m_1} \cdot e^{-i \ 2 \pi \cdot (f_0 + m_1 f_{1}) \ t }\ , \quad E_{samp}(t) \propto \sum _{m_2=-M_2}^{M_2} A^{(2)}_{m_2} \cdot \ \sum _{k=0}^{\infty } \alpha (\tau _k) \cdot \ e^{-i \ 2\pi \cdot (f_0 + f_d + \ m_2 f_{2}) \ (t - \tau _k) } \end{aligned}$$

where $E_{ref}$ is the optical field associated with the reference OFC consisting of $2 \cdot M_1 +1$ optical modes at frequencies $f_{m_1} = f_0 + m_1\cdot f_1$ with corresponding $A^{{\tiny (1)}}_{m_1}$ amplitude. $E_{samp}$ describes the sample OFC after it has traveled through the dispersive media. It consists of $2 \cdot M_2 +1$ modes at frequencies $f_{m_2} = f_0 + m_2 \cdot f_2 = f_0 + m_2\ (f_1 + \Delta f)$. Dispersive media exhibit high absorption and scattering. Their optical response can be described as a distribution of *k* optical paths with different associated delays $\tau _k$, and attenuations $\alpha (\tau _k)$^17^. The transmitted amplitude of the optical paths as a function of the delay corresponds to the photon’s packet Time of Flight (TOF) inside the medium. The sample OFC can be modeled as the superposition of these k paths, as shown in Eq. (1).

When the two OFCs are combined using an optical coupler and photodiodes as square law detectors in a balanced scheme, the resulting interferometric term is the beat note of both signals, as depicted in Fig. 1a. If we consider the lower frequency terms of this new signal, those produced after the beating of the modes with the same order, $m_1 = m_2 = m$, the result is a new comb in the kHz-MHz range around $f_{d}$ with a line spacing, $\Delta f$. Hence, the DC scheme has been able to down-convert the optical information retrieved by the measurement comb around $f_d$, where its acquisition is straightforward^28^, with noiseless amplification^27^. This detected comb can be expressed as:

2 $$\begin{aligned} I_d(t) \propto \sum _{m=-M}^{M} \ \ A^{(1)}_m \cdot A^{(2)}_m \ \cdot \ e^{-i \ 2 \pi \cdot (f_d + \ m \ \Delta f) \ t } \ \sum _{k=0}^{\infty } \ \alpha (\tau _k ) \cdot \ e^{-i \ 2 \pi \cdot (f_0 + f_d + \ m( f_{1} + \Delta f)) \ \tau _k } \end{aligned}$$

with $I_d(t)$ as the corresponding photocurrent after detection. In time domain (TD) techniques^2^, the measurement of the DTOF is based on the convolution of a short pulse or Instrument Response Function (IRF) with the impulse response of the medium. DC-NIRS works in the frequency domain and the detected intensity is formed by a set of lines sampling the frequency response of the dispersive media, as described in Fig. 1b and Eq. (2). The effect of the medium over each frequency translates into an attenuation and phase shift, which can be used to reconstruct the frequency response of the system. Since the frequency response of the medium and its impulse response (or DTOF) are Fourier transform pairs^16,29^, the spectral information sampled with DC-NIRS can be used to infer the DTOF. The maximum sampled frequency determines the temporal resolution of the DTOF reconstruction to $dt = 1/(2 \cdot M \cdot f_{2}))$, and the frequency spacing sets the temporal range of the reconstructed DTOF, $T_{max} = 1/ f_{2}$. The DTOF measures the delay between photons propagating through *k* different paths, this gives information on distance and depth inside the dispersive tissue. Temporal resolution can be related to the spatial resolution of the system as $dr=\Delta t \cdot c/n$, where *c* is the speed of light and *n* is the refractive index of the medium. As the line spacing, $f_{1,2}$, of the OFCs can be freely configured, the resolution and range of this new DC technique can be tuned and adapted to the tissue under test at will and extract its optical properties with spatial localization.

Dynamic changes in the frequency response can also be resolved with DC-NIRS by adjusting the sampling rate of the down-converted comb thanks to the broad catalog of detection and acquisition techniques available in electronics. This opens the door for DTOF temporal evolution analysis, as pictured in Fig. 1c. Another interesting feature of DC-NIRS is a clean and straightforward calibration method to remove the IRF effect. The IRF frequency response can be estimated by measuring without a medium. Performing a simple division between the measured frequency response with the medium and this IRF is equivalent to the deconvolution in the time domain.

### Experimental set-up description

**Figure 2:**
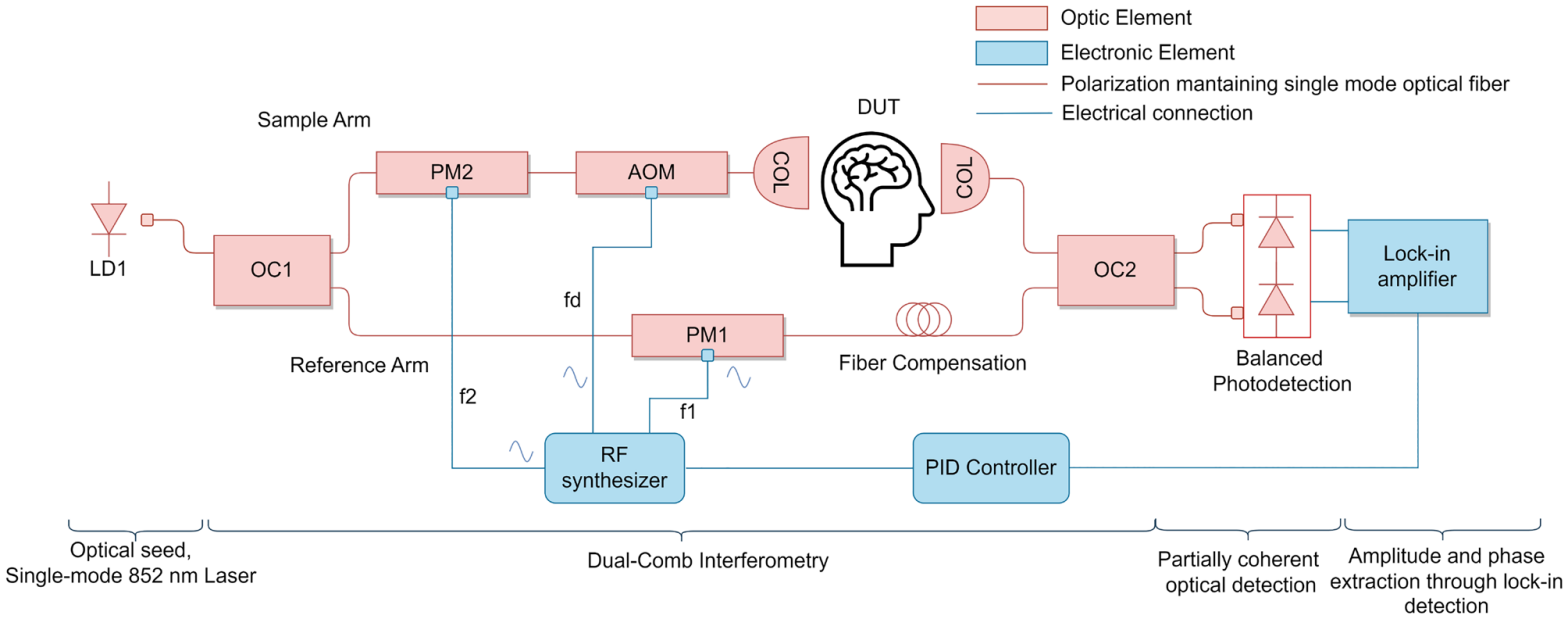
Block diagram of the dual-comb near-infrared spectroscopy system (DC-NIRS). Two optical combs are generated from the same laser source (LD1), whose output power is evenly split by means of an optical coupler (OC1). The reference comb is generated by means of electro-optic phase modulation (PM1) at $f_1$. On the other hand, the sample comb with line spacing $f_2=f_1+ \Delta f$ is generated by phase modulation (PM2), frequency-shifted by an acousto-optic modulator (AOM), and used to illuminate the bio-mimetic phantom and collected back into the fiber through two collimators (COL). Both combs are interferometrically combined by an optical coupler (OC2), photodetected and lock-in amplified to recover the amplitude and phase of each tone. The system is patent pending PCT/ES2022/070176.

**Figure 3:**
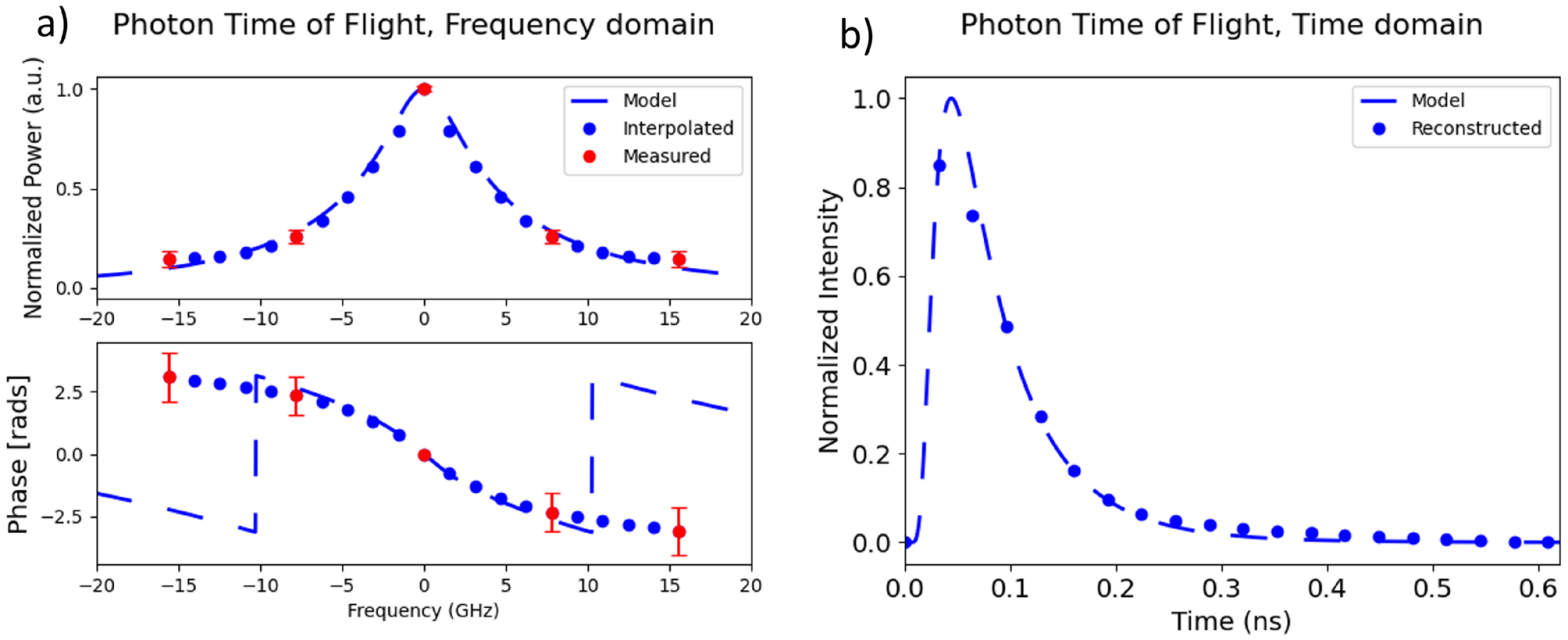
Photon Time-of-Flight, reconstructed and modeled solving the diffusion equation for a finite slab, in (**a**) frequency domain, (**b**) the time domain, the two domains are exchangeable through a Fourier transform. The interpolation on the frequency domain shows the same behavior as the frequency response calculated by the model. The interpolation ratio is 5, increasing the maximum time from 0.13 ns to 0.65 ns. Error bars in the frequency domain measurements show the effect of the attenuation over the phase noise. The phase noise on the central tone is negligible due to the temperature stabilization.The measurements show deviations from the modeled amplitude at high-frequency values due to the effect of the noise floor.

The experiment set-up is aimed at the DTOF reconstruction and measurement of the optical properties in dispersive media by sampling their optical frequency response with dual-comb interrogation. The experimental design, depicted in Fig. 2, is based on an all-fiber, polarization-maintaining (780HP fiber, Thorlabs), electro-optic architecture with a continuous-wave single-mode laser emitting at $\lambda _c$=852 nm and 9 mW (EYP-DFB-0852-00010-1500-BFY12-0005, Eagleyard butterfly laser). This optical seed is independently phase modulated (NIR-MPX800-LN-05, ixblue electro-optic modulator) in both arms of the interferometer to create the reference and sample OFCs. Besides, the sample OFC is frequency-shifted by an acousto-optic modulator (M1205-P80L-1, Isomet) to allow for unambiguous optical detection in the RF domain. The sample arm injects the modulated light into the device under test (DUT) and collects it into a fiber through collimators (Injection collimator, PAF2-A4B, Thorlabs; collection collimator, CFC2-B, Thorlabs). Then, the light that goes through both arms is combined and detected with a balanced photodetector (HBPR-200M-30K-SI-FC, Femto). The DUT is composed of a calibrated bio-mimetic phantom of known optical properties (INO Biomimic optical phantom with $\mu _a = 0.355\,\textrm{cm}^{-1}$, $\mu _s = 13.3\,\textrm{cm}^{-1}$ and $n=1.511$ at $\lambda =850\,\textrm{nm}$) that exhibits varying width that ranges from 3 to 6 mm. As the optical properties are known and the medium’s length is controlled, we can estimate the maximum delay induced by the medium through the solution of the radiative transport equation^30,31^ in transmission for a slab of a fixed width, as we show in Fig. 3, where the modeled frequency response of the medium and its DTOF is contrasted with our measurements. For the 6mm slab width, the DTOF temporal range is estimated to be below 200 ps. This limits the value of the comb teeth separation to sample the DTOF with enough resolution, to a maximum of $2/200\,\textrm{ps} = 10\,\textrm{GHz}$. In the experiment we choose $f_{1}= 7.8\,\textrm{GHz}$ with $\Delta f = 100\,\textrm{kHz}$ and $f_d = 80\,\textrm{MHz}$. For this case, five modes of the sample OFC are recovered after traveling through the dispersive media, $m=\pm 2,\pm 1,0$, setting the frequency range under study to $BW = 2\cdot M \cdot f_{1}=2\cdot 2 \cdot 7.8\,\textrm{GHz} = 31.2\,\textrm{GHz}$. This sets a temporal resolution value of $\Delta t = 32\,\textrm{ps}$. If more detected comb modes are required to evaluate a different sample, the use of a higher optical power laser source and a more efficient optical coupling can be considered.

DC-NIRS needs calibration in order to eliminate the IRF influence. This can be done in a straightforward way with an initial measurement of the amplitude and phase of the tones without the medium (IRF). Then the measurements with the DUT are divided by the measured IRF in the complex plane. To carry out the reconstruction of the DTOF from our experimental spectral data, we carried out standard interpolation and extrapolation processing in order to compensate for the low number of measurement points. As the medium under study is homogeneous and static, the shape of its optical response is known (see Fig. 3a, blue dashed lines). The amplitude shows a quadratic behavior and the phase has small deviations from a linear response. With this information, we performed quadratic spline interpolation and extrapolation of the amplitude and the phase. One key factor in calculating the DTOF, is reconstructing the phase information correctly. Since the phase varies linearly at higher frequencies, the effect of the frequencies selected is minimum on the interpolation error. Amplitude reconstruction can be affected by interpolation. If the line spacing is small there is not enough information and if it is too large, the sampling only will retrieve information about the floor noise. The reconstructed frequency response is presented in Fig. 3a, blue dots. The maximum temporal span of the reconstructed DTOF and its resolution are enhanced thanks to this processing. The span gets extended from 0.13 ns to 0.65 ns and the temporal resolution from $\Delta t = 32\,\textrm{ps}$ to $\Delta t = 27.5\,\textrm{ps}$. In terms of the spatial resolution of the DC-NIRS scheme, it changes from $dr=\Delta t \cdot c/n = 6.5\,\textrm{mm}$ to $\Delta r = 5.5\,\textrm{mm}$. The photons going through the medium travel distances much greater than the width of the slab, up to 4 cm in this case, allowing for the resolved photon paths to have a spatial resolution comparable to, or under the medium’s width.

The interferometric nature of the DC-NIRS technique offers noiseless optical amplification^32^, which enhances the penetration on the sample and compensates for the power lost over the light collection, and also provides a phase reference for absolute phase measurements. In a previous work^27^ with this setup, an optical amplification of 30 dB compared to a non-interferometric approach (without the reference arm) of the comb lines over the noise floor was demonstrated. Additionally, our scheme employs lock-in detection that further enhances the SNR of the system. The Lock-in amplifier was configured to demodulate the frequency response of the medium with a temporal resolution of $70\upmu$s, limited by the value of $\Delta f$.The attenuation of the higher-order frequency terms reduces the SNR, leading to an increase in the error, which affects the amplitude and especially the phase. The decrease in SNR is an effect of the medium response. In order to minimize the effects of the phase noise, an averaging temporal window of 1 second is performed with a sample rate of 14.39 kS/s. Since the medium is homogeneous and static, averaging the frequency response does not imply any loss of information. Our experimental implementation also includes a closed feedback loop to stabilize the system thermally and mechanically^19^. The mismatches between interferometric paths have also been compensated to avoid unwanted effects and increase the quality of the recovered signal.

## Results

### Experimental measurement of the optical properties of a dispersive medium

**Figure 4:**
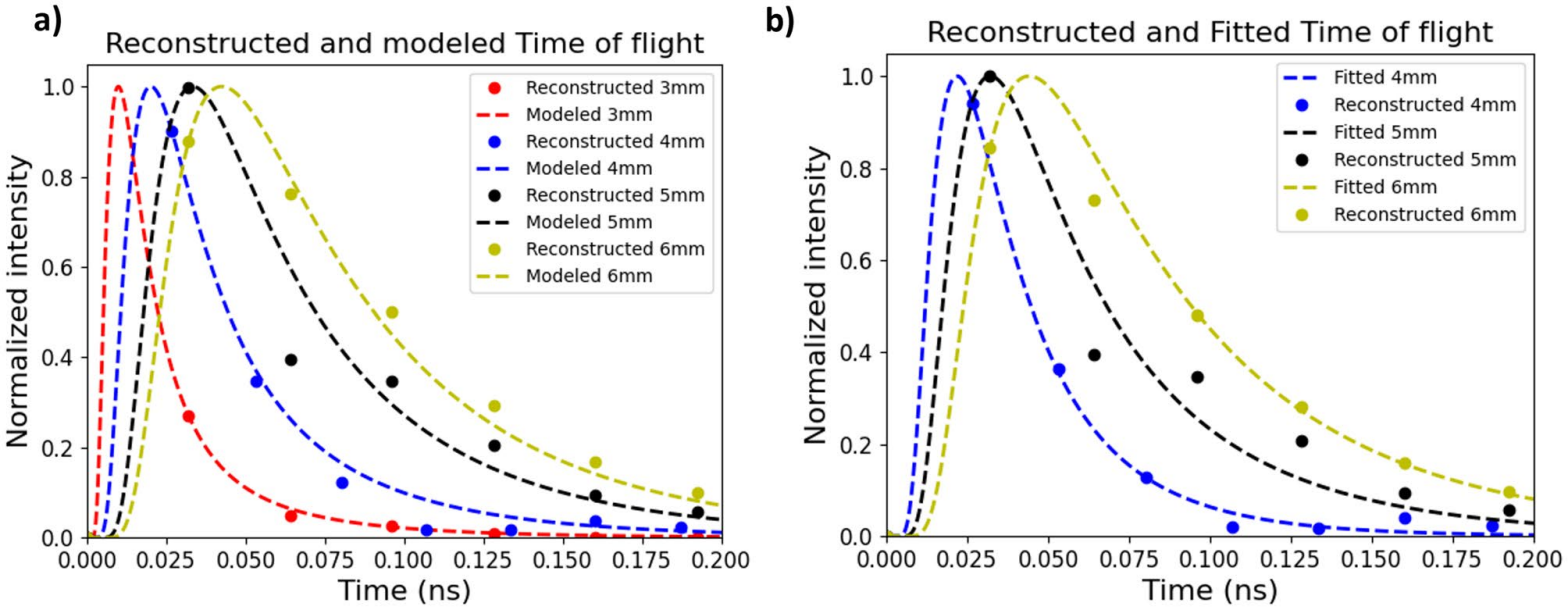
(**a**) DTOF reconstructed for several DUT widths and compared with the model for $\mu _a = 0.355\,\textrm{cm}^{-1}$, $\mu _s = 13.3\,\textrm{cm}^{-1}$. (**b**) Fitted time-resolved diffusion equation solutions for different widths. The reconstructed DTOF in (**a**) and (**b**) contains the same information, where for every width, normalization of the experimental data was made with respect to the maximum of the theoretical model. The part of the DTOF sampled affects the recovery of the optical properties since the tail contains information of $\mu _a$ and the width is used to infer $\mu _s$. For the 3 mm slab, the fit does not converge, and boundary conditions were achieved, it was omitted for clarity in (**b**).

The DC-NIRS technique was experimentally tested to extract the optical properties of the reference medium (INO Biomimic optical phantom with $\mu _a = 0.355\,\textrm{cm}^{-1}$ and $\mu _s = 13.3\,\textrm{cm}^{-1}$). Its frequency response was sampled for different medium widths that cover the range from 3 to 6 mm. Figure 4a shows the reconstructed DTOF from our measurements (dots), along with the solution of the TOF diffusion equation in transmission^30,31^ for the different widths (dashed line). The results show a broadening of the photon resolved time-of-flight as the medium width increases. The reconstructed data is consistent with the model calculations for the DUT. IRF influence in the reconstructed data is compensated by comparing the measurements to the calibration frequency response without the DUT, which validates DC-NIRS as a suitable technique to recover photon path distributions of dispersive media.

**Table 1:** The estimated optical properties for the different sample widths.

L (mm)	$\mu _{a}[\textrm{cm}^{-1}]$	$\mu _{s}[\textrm{cm}^{-1}]$	R squared (%)	z-score $\mu _a$	z-score $\mu _s$
Real	0.355	13.30	–	–	–
3	0.250	5.69	–	2.19	6.15
4	0.734	14.19	98.3	1.94	1.03
5	0.358	12.91	99.2	0.04	1.05
6	0.327	13.41	99.3	0.82	0.91

The real values are compared with the values fitted. In the case of 3mm boundary conditions are achieved.

To estimate the optical properties of an unknown medium, the measurements can be fit to the model, where the fitting parameters are $\mu _a$ and $\mu _s$ since the rest of them are defined or controlled by the experiment. Minimization of the mean squared error of the measurement versus the model was performed using a Nelder-Mead^33^ algorithm in Python. The fitted results of the measured DTOFs to the diffusion equation $\mu _a$ and $\mu _s$ are shown in Fig. 4b. In both figures, the reconstructed points (dots) are normalized to the maximum value of the modeled DTOF (dashed lines), this facilitates the comparison of each DTOF. Absorption modifies the transmission of each optical path since longer optical paths are more attenuated than shorter ones. This has an impact on the DTOF slope. On the other hand, the scattering coefficient modifies the width of the DTOF by increasing the number of optical paths on both sides of the distribution^9,16^. Since the scattering coefficient $\mu _s$ determines the photon’s DTOF width, the interrogation of the tail of the distribution does not suffice to accurately estimate this parameter of the sample, showing better results for larger widths, in which the positive slope area of the DTOF is sampled and the width can be estimated. For shorter widths, as is the case for the 3mm medium width, the temporal resolution is not enough to sample sufficient information of the distribution and perform a good fit of $\mu _s$ and $\mu _a$. For the media interrogated in this experiment, the best results were achieved for 5mm and 6mm widths. The calculated $R^2$ value determines the difference between the fitted DTOF and the modeled one. With this value, we can quantify the deviation of the DTOF shape from the theoretical model for the known values. The values of fitted $\mu _a$, $\mu _s$ and $R^2$ for different widths, are shown in Table 1.

Medium lengths of 3 and 4 mm imply a maximum TOF (where 90$\%$ of the relevant photon paths are resolved) under 100 ps with a distribution peak time shorter than the temporal resolution. This translates into a DTOF sampled by a small number of points and only at the distribution’s tail. The high values of $R^2$ show that the fitted DTOFs have slight deviations from the modeled ones, but the certainty of the optical properties recuperation varies since small errors in the measurements can set the fit point at different $\mu _a$ and $\mu _s$, especially for a smaller number of sample points.Analyzing the z-scores $\mu _a$ and $\mu _s$, we notice that for shorter lengths (3 and 4 mm), these values are further from one, indicating a less accurate estimation of the optical properties. The fitting, for the 3 mm case, does not converge, and boundary conditions were achieved.

Relevant measurements were achieved when the time of the maximum transmission is greater than the temporal resolution of the system, so we can sample information on the width and tail of the distribution. For the 6 mm slab a deviation of $8 \%$ and $0.88 \%$ from the real values of the medium’s optical properties $\mu _a$ and $\mu _s$ was achieved.The z-scores for $\mu _a$ and $\mu _s$ in for the 5 and 6 mm slab indicate the number of standard deviations from the mean value, affirming the accuracy of our measurements with values under one standard deviation, implying a good fit and less variation from the true values, as expected. Although absorption and scattering coefficients affect different parts of the distribution, they are strictly related, and the convergence of the fitted values is heavily influenced by the measured DTOF shape. The difference between the fitted and real values as well as the $R^2$ value shows that the quality of the recovered DTOFs increases as the number of relevant sampled points on the DTOF increases accordingly in the relevant parts of the distribution as the tail, and the positive slope area.

## Conclusion

A novel technique for NIRS based on Dual Combs, DC-NIRS, has been presented and experimentally validated. The capabilities of the new DC-NIRS scheme for the assessment of optical properties in dispersive media were evaluated with remarkable results. We demonstrated the use of Dual-comb interferometry to interrogate dispersive samples with parallel amplitude and phase demodulation. The technique retrieves the frequency response of the medium with a single measurement, without the need to sweep any parameters. DC generation through electro-optic phase modulation offers high adaptability since the temporal (32 ps) and spatial resolution (6.5 mm) can be easily configured. Heterodyne gain combined with lock-in amplification allows for an increased SNR over the photodetected signal, enhancing the recuperation of the low transmission photon paths. Absolute values of the absorption coefficient, the scattering coefficient, and the DTOF were measured for a static biomimetic phantom for several medium widths. For the 5 mm and 6 mm slab, where the DC-NIRS mode separation and span are optimal, we obtain small deviations ($0.85 \%$ and $0.88 \%$ for $\mu _a$ and $\mu _s$ respectively) from the real values. All of these factors set the foundation for a new, faster, and more versatile NIRS technique that also features enhanced temporal and spatial resolution, opening the door to the study of fast hemodynamics present in biological samples, such as the brain, thanks to the potential of the scheme to recover dynamic DTOF reconstruction with fine temporal resolution. We have tested the viability of the technique with experimental results for a static biomimetic phantom confirming the capacities of the DC-NIRS technique for diffuse medium interrogations, matching other techniques such as FD-NIRS, TD-NIRS, and iNIRS with a configurable, agile and fast new approach.This lays the groundwork for functional applications that can leverage the capabilities showcased in this study, contributing to the development of an innovative fNIRS technique. The system is patent pending PCT/ES2022/070176.

## Data Availability

The datasets generated during and/or analyzed during the current study are available from the corresponding author upon reasonable request.
